# Complete genome sequence of *Bordetella parapertussis* strain 400431-b, isolated from a protracted course of whooping cough in Austria, 2023

**DOI:** 10.1128/MRA.00976-23

**Published:** 2023-11-29

**Authors:** Maria João Cardoso, David Rouhani, Adriana Cabal Rosel, Beatriz Daza Prieto, Miriam Hopfgartner, Anna Stöger, Petra Hasenberger, Silke Stadlbauer, Tobias Moesenbacher, Patrick Hyden, Ursula Wiedermann, Daniela Schmid, Werner Ruppitsch

**Affiliations:** 1 Institute of Medical Microbiology and Hygiene, Austrian Agency for Health and Food Safety, Vienna, Austria; 2 ECDC Fellowship Programme, Public Health Microbiology path (EUPHEM), European Centre for Disease Prevention and Control (ECDC), Stockholm, Sweden; 3 St. Anna Children’s Hospital, Vienna, Austria; 4 Institute of Chemical, Environmental, and Bioscience Engineering, Research Area of Biochemical Technology, Technical University of Vienna, Vienna, Austria; 5 Institute of Specific Prophylaxis and Tropical Medicine, Center for Pathophysiology, Infectiology and Immunology, Medical University of Vienna, Vienna, Austria; 6 Division of Infection Diagnosis and Infectious Disease Epidemiology, Center of Pathophysiology, Infectiology and Immunology, Medical University of Vienna, Vienna, Austria; 7 Department of Biotechnology, University of Natural Resources and Life Sciences, Vienna, Austria; University of Maryland School of Medicine, Baltimore, Maryland, USA

**Keywords:** whooping cough, WGS, antimicrobial resistance, *Bordetella*, genomics

## Abstract

Here, we report the complete genome of *Bordetella parapertussis* strain 400431-b isolated from a nasopharyngeal swab from a 4-year-old patient presenting with a protracted course of whooping cough, vaccinated with three doses of diphtheria-tetanus-acellular pertussis-hepatitis B-poliomyelitis-*Haemophilus influenzae* type b vaccine.

## ANNOUNCEMENT

*Bordetella parapertussis* (Bpp) 400431-b was isolated from a 4-year-old patient and vaccinated with three doses of diphtheria-tetanus-acellular pertussis-hepatitis B-poliomyelitis-*Haemophilus influenzae* type b vaccine, showing a protracted clinical course of whooping cough. The nasopharyngeal swab was plated onto Bordetella Selective Medium (Oxoid, Waltham, MA, USA) and incubated 48 hours at 37°C. Presumptive *Bordetella* spp. colonies were subcultivated on blood agar (COS, Biomérieux, Marcy-l'Étoile, France) and identified by matrix-assisted laser desorption/ionization time-of-flight mass spectrometry (Microflex LT/SH, MBT Compass IVD4.2, Flex Analysis v3.4, Bruker, Billerica, MA, USA).

Antimicrobial susceptibility testing was performed as recommended by the European Committee on Antimicrobial Susceptibility Testing (EUCAST) with minimum inhibitory concentration (MIC) gradient strips containing erythromycin, azithromycin, clarithromycin, and trimethoprim-sulfamethoxazole (ETEST, Biomérieux, Marcy-l'Étoile, France) on Mueller-Hinton agar + 5% blood + 20 mg/L β-NAD (Biomérieux, Marcy-l'Étoile, France). Due to the lack of EUCAST breakpoints, susceptibility results were compared with strain FR6242 (1).

Genomic DNA was isolated from cells grown on COS, for 48 h at 37°C. The MagAttract HMW (Qiagen, Hilden, Germany) and the Wizard HMW (Promega, Madison, WI, USA) DNA Extraction Kits were used for Illumina and Oxford Nanopore sequencing, respectively. Sequencing libraries were prepared using the DNA preparation (M) kit (Illumina, San Diego, CA, USA) and the rapid barcoding kit SQK-RBK004 [Oxford Nanopore Technologies (ONT), Oxford, UK]. Libraries were 2 × 150 bp sequenced on NextSeq 2000 (Illumina, San Diego, CA, USA) as described (2) and with FLO-MIN114 R10.4.1 SpotON flow cell on a GridION (ONT, Oxford, UK). Default parameters were used for all software unless otherwise specified. A total of 3,146,612 Illumina reads with average length of 142 bp were quality controlled using FastQC v0.11.9.12 (3). A total of 293,539 Nanopore reads (N50: 15,211 bp) were obtained using Guppy v6.3.8 (4) in super-accurate basecalling mode and filtered using Filtlong v0.2.1 (parameters: -t 400000000 min_length 1000 --keep_percent 95). Assembly was performed using Flye v2.9.2-b1786 (5). Draft assembly was polished using Medaka_consensus v.1.8.0 with model r1041_e82_260bps_sup_g632 followed by Polypolish v.0.5.0 (6), which resulted in one contig with 92-fold average coverage, a genome size of 4.8 Mb, and a GC content of 68.1% (Table 1). Burrows-Wheeler Aligner Maximal-Exact-Matches algorithm (BWA-MEM) (7) and SAMtools v.1.17 (8) were used to manually inspect reads at the contig ends to check circularity of the final assembly.

**TABLE 1:** Characteristics of *B. parapertussis* 400431-b

Parameter	Finding/strain/accession	
Total genome size (bp)	4,773,379 (circular)	
No. of contigs	1	
Hybrid assembly genome coverage (×)	92	
No. of raw short reads	3,146,612	
No. of raw long reads	293,539	
Long-read fragment N50 (bp)	15,211	
GC content (%)	68.1	
Total no. of genes	4,521	
Total no. of coding sequences	4,223	
No. of RNA genes	67	
No. of complete rRNAs (5S, 16S, 23S)	3, 3, 3	
No. of tRNAs	54	
No. of noncoding RNAs	4	
Total no. of pseudogenes	231	
No. of CRISPR/Cas elements	0	
No. of insertion sequences IS1001 (identity >99.0%)	20 (with 4 to 7 substitutions)	
No. of insertion sequences IS1002 (identity >98.6%)	9 (with 11 to 13 substitutions)	
No. of composite transposons_IS1001	3 (with 5 to 7 substitutions)	
No. of plasmids	0	
No. of phages	0	
Sequence type	19	
Virulence genes	Present: - Fimbrial precursors (*fim2, fim3*) - Pertussis toxin promoter (*ptxP*) - Pertussis toxin (*ptxA, ptxB, ptxC, ptxD, ptxE*) - Filamentous hemagglutinin (*fhaB*) - Dermonecrotic toxin (*dnt*) - Adenylate cyclase (*cyaA*) -Pertactin (*prn*)^*a*^	Absent: - Tracheal colonization factor (*tcfA*)
Efflux pumps	*adeF* (two sequences) *qacG*	
MIC trimethoprim-sulfamethoxazole (concentration 1/19) (mg/L)	6	
MIC erythromycin (mg/L)	1.5	
MIC clarithromycin (mg/L)	1.5	
MIC azithromycin (mg/L)	0.75	
cgMLST reference genome	Strain 12822: NC_002928.3	
cgMLST query genomes	Bpp5: NC_018828.1, NC018830.1; H904: NZ_CP016342.1; H889: NZ_CP018897.1; A747: NZ_CP020655.1; B337: NZ_CP020654.1; D577: NZ_CP020652.1; E738: NZ_CP020648; I440: NZ_LS483424.1; NCTC10520: NZ_LS483424.1; NCTC10524: NZ_LR134329.1; Bpp01: NZ_AP019378.2; A005: NZ_CP025070.1; B114: NZ_CP025068.1; B149: NZ_CP025071.1; B160: NZ_CP025072.1; B271: NZ_CP025067.1; F585: NZ_CP024174.1; E762: NZ_CP019932.1; E843: NZ_CP019931.1; NCTC10522: NZ_LR590480.1; H100: NZ_CP043165.1; H101: NZ_CP043164.1; H102: NZ_CP043163.1; H104: NZ_CP043162.1; H105: NZ_CP043161.1; H242: NZ_CP043159.1; H314: NZ_CP043155.1; H554: NZ_CP043145.1; H555: NZ_CP043144.1; H580: NZ_CP043140.1; H581: NZ_CP043139.1; I133: NZ_CP043121.1; I139: NZ_CP043120.1; FDAARGOS_1540: NZ_CP085972.1; FDAARGOS_1541: NZ_CP085971.1.	

^*a*^ *prn* promoter is absent; *prn* is non-functional due to deletion in position 1893.

AMRFinderPlus v3.11.2 (9), MOB-suite v3.1.4 (10), MobileElementFinder v1.0.5 (11), and VFDB v2020-Feb-28 (12) were used for the detection of antimicrobial resistance genes, virulence genes, and mobile genetic elements.

The NCBI Prokaryotic Genome Annotation Pipeline (PGAP) v6.6 (13) identified 4,521 genes, 4,223 coding sequences, and 67 RNA genes (Table 1). An *ad hoc* core genome multilocus sequence typing scheme comprising 3,479 core targets was created using the Target-Definer v1.5 from SeqSphere + v8.5.1 (Ridom, Münster, Germany), using strain 12822 (14) as reference and 35 Bpp query genomes from GenBank (Table 1 ). Comparison of Bpp 400431-b with all available genomes from GenBank showed 37, 16, and 10 allelic differences to strains 12822, FR6242 (1), and J456 (15), respectively.

Bpp 400431-b carried *bla_BOR-1_* , three efflux pumps, and had 50-, 12-, and 8-fold higher MICs for trimethoprim-sulfamethoxazole, erythromycin, and clarithromycin than strain FR6242, respectively.

## Data Availability

This whole genome project was deposited in NCBI GenBank database under the accession number CP134883, BioProject accession number PRJNA1013794, and BioSample accession number SAMN37311000. This is the first version of this genome. Illumina raw sequence reads have been deposited in the Sequence Read Archive (SRA) under the accession number SRR26175772 and Oxford Nanopore reads under SRA accession number SRR25937767.

## AFTER EPUB

*[This article was published on 29 November 2023 with an error in Table 1. The error was corrected in the current version, posted on 12 December 2023.]*
